# Formation of Microporous Poly Acrylonitrile-Co-Methyl Acrylate Membrane via Thermally Induced Phase Separation for Immiscible Oil/Water Separation

**DOI:** 10.3390/molecules29102302

**Published:** 2024-05-14

**Authors:** Linli Tan, Yuqi Wang, Mingpu Li

**Affiliations:** 1 College of Intelligent Systems Science and Technology, Hubei Minzu University, Enshi 445000, China; 2 Key Laboratory of Green Manufacturing of Super-Light Elastomer Materials of State Ethnic Affairs Com, Enshi 445000, China

**Keywords:** poly(acrylonitrile-co-methyl acrylate), membranes, thermally induced phase separation, oil/water separation

## Abstract

An interconnected sponge structure and porous surface poly (acrylonitrile-co-methyl acrylate) (P(AN-MA)) microfiltration membranes (MF) were fabricated via thermally induced phase separation (TIPS) by using caprolactam (CPL), and acetamide (AC) as the mixed diluent. When the ternary system was composed of 15 wt.% P(AN-MA), 90 wt.% CPL, and 10 wt.% AC and formed in a 25 °C air bath, the membrane exhibited the highest water flux of 8107 L/m^2^·h. The P(AN-MA) membrane contained hydrophobic groups (-COOCH_3_) and hydrophilic groups (-CN), leading it to exhibit oleophobic properties underwater and hydrophobic properties in oil. The membrane demonstrates efficient separation of immiscible oil/water mixtures. The pure water flux of the petroleum ether/water mixture measured 870 L/m^2^·h, and the pure oil flux of the petroleum tetrachloride/water mixture measured 1230 L/m^2^·h under the influence of gravity. Additionally, the recovery efficiency of diluents through recrystallization was 85.3%, significantly reducing potential pollution and production costs.

## 1. Introduction

Presently, membrane separation technology is recognized as an environmentally friendly separation process that has found widespread application in environmental, electronic, food, and pharmaceutical production, as well as in oil/water separation [1,2,3,4,5]. In recent years, significant efforts have been focused on fabricating novel membrane structures and achieving excellent membrane performance through a simple and repeatable method. Currently, the thermally induced phase separation (TIPS) method has emerged as a leading technique for preparing polymer membranes [2,6,7,8]. This is attributed to its ease of controllability, low tendency toward defect formation, repeatability, and diverse pore structures. Furthermore, membranes fabricated using TIPS exhibit higher mechanical strength, greater porosity, a narrower pore size distribution, and better repeatability than those produced using the non-solvent-induced phase separation (NIPS) method [6,9,10]. Importantly, the TIPS method enables the preparation of polymer membranes for materials that do not have a suitable solvent at room temperature, such as high-density polyethylene (HDPE) [11], ultrahigh molecular-weight polyethylene (UHMWPE) [12], polyphenylene sulfide (PPS) [13], poly(ethylene chlorotrifluoroethylene) (ECTFE) [14], cellulose triacetate [15], and polyamide 6 [10]. During the TIPS process, a homogeneous solution is obtained by dissolving polymer in a high-boiling-point and low-volatility solution at a high temperature above the melting temperature of the homogeneous solution, and then cooling or quenching the solution to induce phase separation and precipitation of the polymers [2,10,16]. Finally, the diluent is removed by the appropriate extractants to yield microporous and ultrafiltration membranes. During the removal of thermal energy to induce phase separation, the membrane structures are determined by the properties of the homogeneous solution. The membranes formed by different phase separation pathways exhibit distinct morphologies [8,15,17,18,19]. Membranes formed through the liquid-liquid (L-L) separation pathway typically exhibit a porous honeycomb-, cellular-, or sponge-like morphology. In contrast, membranes formed via the solid–liquid (S-L), liquid-solid (L–S), or solid–solid (S-S) separation pathways display spherulitic, needle-like, or sheet-like pore structures, depending on the interactions between the polymer and the solvent, the composition of the polymer solution, and the thermal driving force. Hence, thermodynamic and kinetic parameters can modulate the pore structures and morphology of polymer membranes. 

Polyacrylonitrile (PAN) was used as the membrane matrix due to its outstanding thermal stability, chemical resistance, and excellent mechanical properties. Consequently, PAN and PAN-copolymer membranes have been widely applied in water treatment [20,21], oil/water separation [22], support layers of graphite oxide film [23] and CO_2_ capture [24], nano-filtration (NF) [25] or reverse osmosis (RO) membranes [26]. To the best of our knowledge, commercial PAN membranes are still fabricated using the NIPS method, resulting in typical finger-like structures [23,26]. These issues have significantly impacted the strength, performance, and inherent limitations of controlling pore structures, thereby affecting the application prospects of PAN membranes.

However, despite the TIPS method being in development for over 30 years [27], there are fewer literature references for PAN membranes fabricated using TIPS compared with poly(vinylidene fluoride) (PVDF) membranes. The primary challenge lies in selecting a suitable solvent for PAN via TIPS. In 2010, Jiang et al. [28] utilized dimethylsulfoxide (DMSO) to fabricate PAN microporous membranes through a viable freeze method. However, the fabrication required conditions within a freezer set at temperatures below minus ten degrees Celsius (−10 °C), thereby escalating the difficulty of membrane fabrication. In 2012, Xu et al. extensively described the interactions between PAN and various conventional solvents [29] (such as dimethyl sulfone (DMSO_2_), DMSO, ethylene carbonate (EC), propylene carbonate, N,N-dimethyl formamide, N,N-dimethyl acetamide) through theoretical calculations and experimentation. They concluded that the crystallizable diluent DMSO_2_ was the only suitable option for preparing porous PAN membranes via TIPS [9,18,30,31,32]. Although they observed numerous pore morphologies (such as cellular-like, sheet-like, or needle-like pores), the average water flux was very low, and subsequent studies primarily focused on hierarchically porous carbon membranes [31,33].

In 2016, Han et al. utilized the copolymer poly (acrylonitrile-co-methyl acrylate) (P(AN-MA)) to fabricate PAN-based membranes using the TIPS method, with the mixed diluents of γ-butyrolactone (γ-BA)/glyceryl triacetate (GTA) and ethylene carbonate ester (EC)/triethyl citrate (TEC). The addition of methyl acrylate not only reduces the interactions and polarity of the PAN-based copolymers [34,35] but also retains the advantage of the nitrile group (-CN). However, similar to previous findings, the water fluxes remained very low; the maximum flux was only 80 L/m^2^·h [36].

Building on these studies, we selected the crystallizable and water-soluble diluent caprolactam (CPL) and the additive acetamide (AC) to prepare P(AN-MA) microporous membranes using the TIPS method. The resulting P(AN-MA) microporous membranes exhibit complete macroscopic and symmetric cross-sectional morphologies with uniformly distributed spongy pores. Due to the addition of methyl acrylate, the P(AN-MA) exhibits hydrophobic and super-oleophilic properties, resulting in the appearance of water droplets and oil droplets on the membrane surface with contact angles of 115° and 0°, respectively. The P(AN-MA) membrane, with its high permeability, will find widespread applications in water treatment and oil/water separation processes.

## 2. Results and Discussion

### 2.1. Phase Diagram of P(AN-MA)/CPL/AC Systems

**Figure 1:**
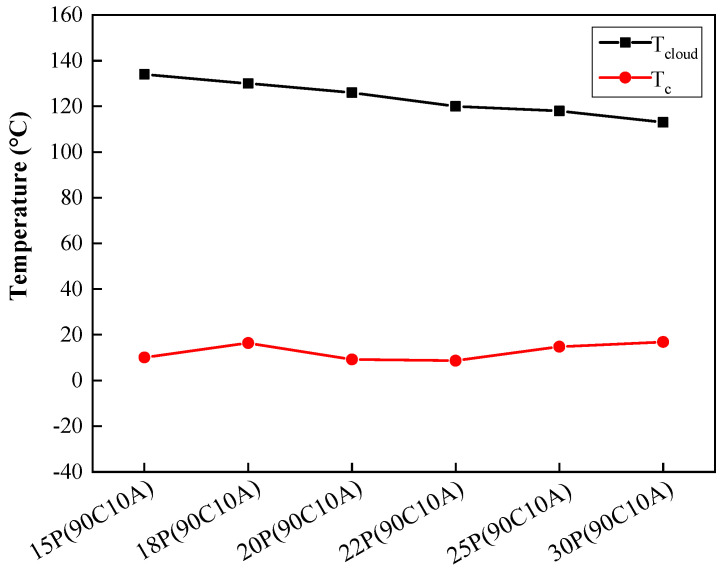
Phase diagram of the P(AN-MA)/CPL/AC ternary system: ■ indicated the cloud points (T_cloud_), ● indicated the dynamic crystallization temperature (Tc).

The phase diagram of P(AN-MA)/CPL/AC exhibited both stable and metastable states, as depicted in Figure 1. Cloud points (T_cloud_) and dynamic crystallization temperature (T_c_) are generally assumed to be the upper and bottom boundary of the L-L phase separation region, respectively. T_cloud_ and T_c_ divide the phase diagram into three regions: a one-phase polymer/solution liquid region, a liquid–liquid (L-L) phase separation region, and a solid region [8,15]. As the P(AN-MA) content increased, the L-L phase separation region narrowed within a fixed diluent composition. In fact, the relative interaction strength between polymer and diluent determined the specific phase separation process (e.g., L-L, S-L, L-S, S-S demixing). It could be assessed by Hansen’s parameter (δ), which included a dispersion force component (δ_d_), polar component (δ_p_), and hydrogen bonding component (δ_h_) [17,18,19]. The polar polymer had similar values of δ and close values of δ_p_ with solvent, which means that they have good compatibility. Additionally, Δδ_12_ was used to calculate the interaction between polymer and solvent. A smaller value of Δδ_12_ indicates a good compatibility between the polymer and solvent. Hansen’s parameters δ and δ_12_ can be directly calculated; the various solubility parameters were listed in Table 1. We can see that P(AN-MA)-CPL (Δδ_12_ = 11.78 MPa^0.5^) had stronger interactions than P(AN-MA)-AC (Δδ_12_ = 267.60 MPa^0.5^). Hence, AC can be used to adjust the interaction strength between P(AN-MA) and CPL. Experimentally, it was found that 10 wt.% AC represents the highest concentration miscible with P(AN-MA)/CPL at 140 °C, leading to phase separation during the cooling process, along with the crystallization of P(AN-MA), CPL, and AC.

### 2.2. Effect of P(AN-MA) Concentration on the Morphology of Membranes

**Figure 2:**
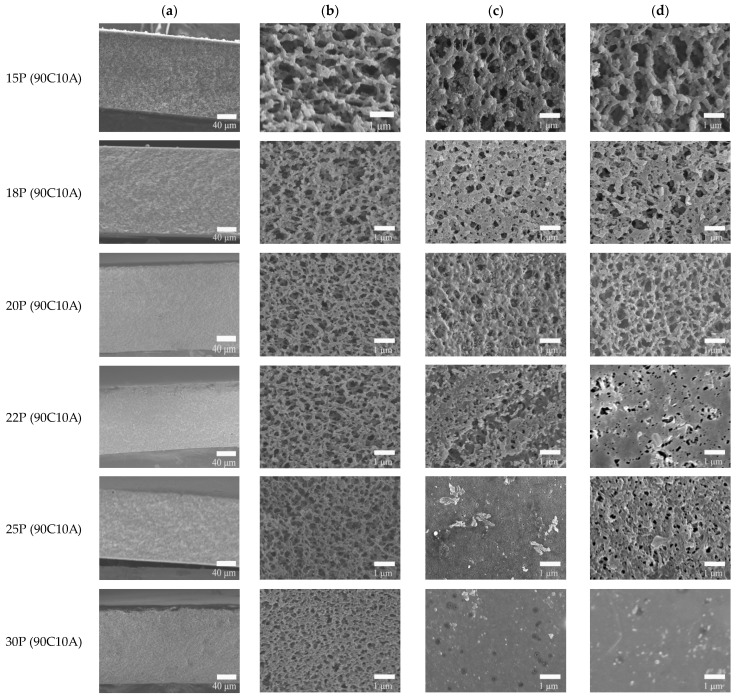
SEM images of P(AN-MA) membranes prepared with 10 wt.% CA in the mixed diluent: (**a**) cross-section (250×), (**b**) enlarged cross-section (10,000×), (**c**,**d**) top and bottom surface (5000×), respectively. The P(AN-MA) content in the ternary system from 15 wt.% to 30wt.%. (25 °C air bath).

**Figure 3:**
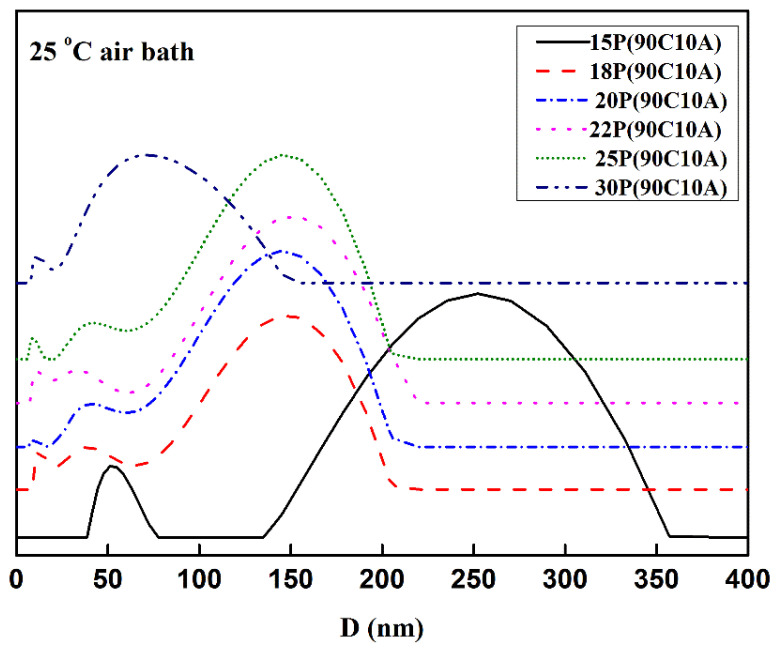
The pore size distribution required to keep the AC content constant at 10 wt.%, P(AN-MA) membrane prepared with various polymer concentration (25 °C air bath).

Experimentally, 10 wt.% of AC is the maximum miscible with P(AN-MA)/CPL at 140 °C. Our research focused on the P(AN-MA) concentration and cooling rate’s influence on the morphology of the membranes. As shown in Figure 2, all the membranes exhibit a symmetric morphology filled with interconnected sponge pores. Upon increasing the P(AN-MA) concentration from 15 wt.% to 30 wt.% in the ternary system, the pore size decreases from 0.25 to 0.07 μm, as depicted in Figure 3.

Moreover, the membrane surface pore size decreased significantly when the polymer content reached 30 wt.%. At this point, the membrane had nearly no discernible pore size. This phenomenon is attributed to the narrowing of the L-L separation regions and the increase in the viscosity of the P(AN-MA) solution. Simultaneously, the bottom of the nascent membrane experienced a lower cooling rate than the top, resulting in larger pore sizes on the bottom surface compared to the top surface.

### 2.3. Effect of Cooling Rate on the Morphology of Membranes

**Figure 4:**
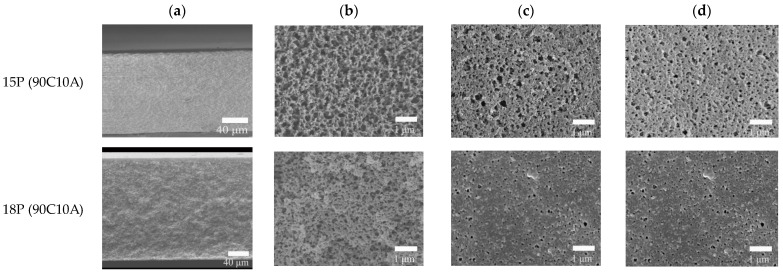
SEM images of P(AN-MA) membranes prepared with 10 wt.% CA in the mixed diluent: (**a**) cross-section (250×), (**b**) enlarged cross-section (10,000×), (**c**,**d**) top and bottom surface (5000×), respectively. The P(AN-MA) content in the ternary system from 15 wt.% to 18wt.%. (25 °C water bath).

**Figure 5:**
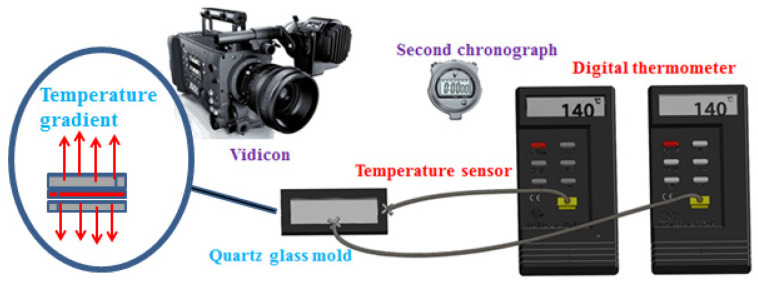
Schematic illustration of the homemade device that recorded the actual cooling process.

**Figure 6:**
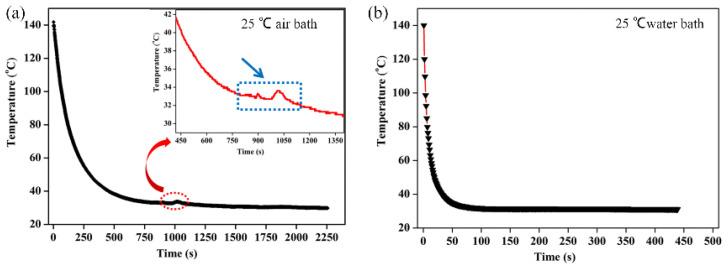
Temperature changes of 15P(90C10A) solution at 25 °C air (**a**) or water (**b**) bath.

The cooling rate plays a fundamental role in controlling the morphologies of membranes in the TIPS method. Figure 4 displays the morphologies of P(AN-MA) membranes prepared under different cooling conditions (25 °C air or water bath). Upon comparing Figure 2 with Figure 4, it becomes evident that all the membranes exhibit symmetrical morphology with interconnected sponge pores, while the pore size decreases as the cooling rate increases. Furthermore, the surface of P(AN-MA) membranes becomes denser when prepared in a 25 °C water bath. To accurately capture the real cooling rate of the P(AN-MA)/CPL/CA ternary system, two temperature sensors were utilized to measure the inside and outside temperatures of the mold, as depicted in Figure 5. This approach aims to provide a thorough understanding of the cooling rates under different conditions and their impact on membrane formation. Vidicon was adopted to record the changes presented by the second chronograph and digital thermometer. The P(AN-MA) nascent solidification membranes can be obtained through non-isothermal and isothermal cooling crystallization approach. In the actual membrane fabrication via TIPS, isothermal phase separation behavior was the most prevalent [27,28], so we adopted the manmade device to measure actual P(AN-MA) solutions’ cooling rate in a 25 °C air bath or water bath. It will be better to reflect the temperature change in relation to the cooling time and understand the relationship between the cooling rate and membrane morphologies. Figure 6 showed the temperature changes of the 15P(90C10A) solution in a quartz glass mold inside and outside, in a 25 °C air and water bath of isothermal cooling crystallization. Compared to the 15P(90C10A) solution’s actual cooling rate, nascent membrane demonstrated a rapid cooling rate in a 25 °C water bath. The mold and nascent membrane solution’s initial average cooling rate was 29 °C/min in a 25 °C air bath; this was less than the initial average cooling rate, which was 480 °C/min in a 25 °C water bath. During the isothermal cooling crystallization process, the cooling rate decreased over time. The comparison of the cooling rates between the water bath and air bath provided valuable insights. The rapid decrease in temperature from 140 °C to 60 °C within 3.3 s in the water bath, as opposed to 155 s at the air bath to reach the same temperature, highlights the significant difference in cooling rates. This supports our understanding that low cooling rates prolong the L-L phase separation time [31,33]. Additionally, it is noted that the viscosity of the P(AN-MA)/CPL/AC solution decreases as the temperature increases [9,29]. Consequently, a lower cooling rate is advantageous for prolonging the growth period of liquid droplets and mitigating the increase in the solution’s viscosity. As we know, crystallization is an exothermic process, and the crystallization exothermic peak could be detected at temperatures near to 28.3 °C, as shown in Figure 6a. This means that the device used to record the actual cooling process is very sensitive. Meanwhile, the water thermal capacity is higher than that of the air, so in Figure 6b, we cannot detect the crystallization exothermic peak in a water bath.

**Figure 7:**
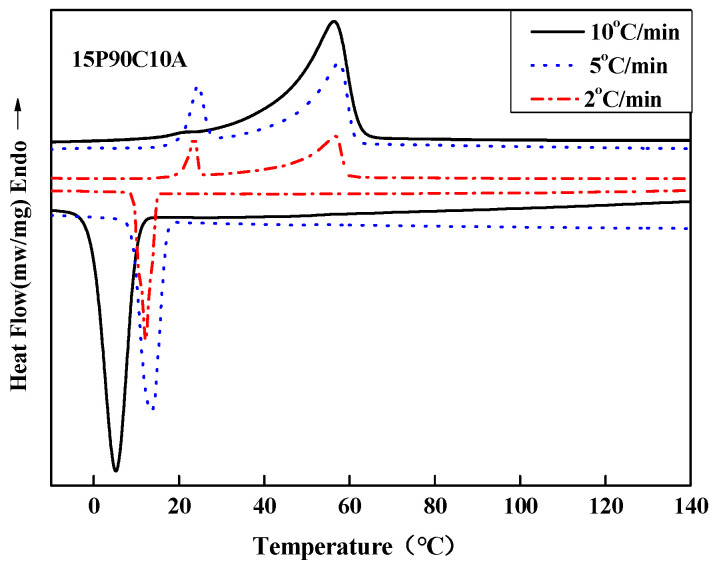
Crystallization and melting behaviors of 15P(50C50C) mixture at different cooling rates. The solid lines represent 10 °C/min, the dotted lines represent 5 °C/min, and the dashed-dot lines represent 2 °C/min.

**Figure 8:**
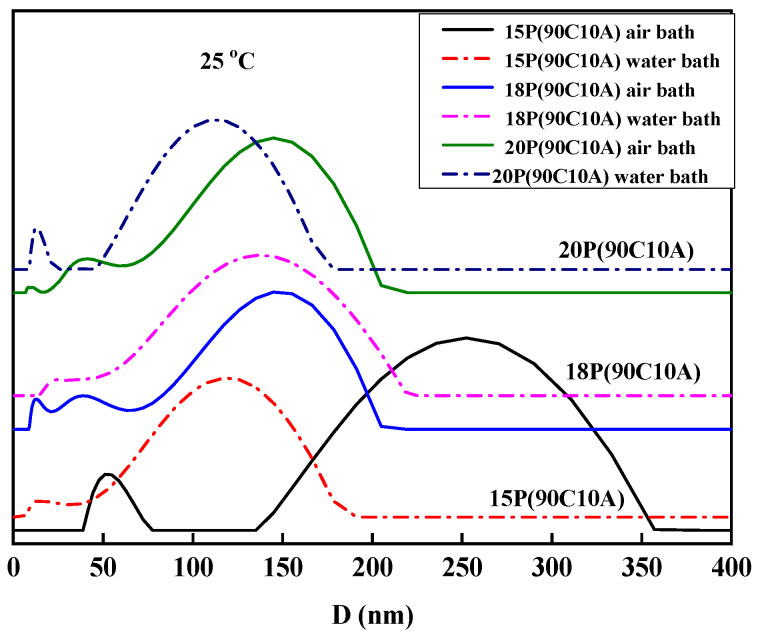
The pore size distribution of keeping AC content constant at 10 wt.%, P(AN-MA) membrane prepared with various polymer concentration (25 °C air and water bath).

To further demonstrate the influence of the cooling rate on the polymer solution, we measured the crystallization and melting behaviors of 15P(90C10A) mixture in the non-isothermal crystallization process at different cooling rates (10, 5, 2 °C/min, respectively), as shown in Figure 7. The onset of the exothermic peak during the cooling process was taken as the non-isothermal crystallization temperature. It can be seen that the initial T_c_ of the samples decreased progressively with the increase in cooling rate, while the endothermic peak changed from one peak to two peaks with the decrease in the cooling and re-melting rate. This meant that the polymer solution had a sufficient L-L phase separation period. The polymer-rich continuous phase and poor phase droplet were fully separated and became two distinct phases at a low cooling rate. So, when re-melting, the polymer solution had two obvious melting peaks. All of the results show that a low cooling rate is better for prolonging the L-L phase separation period. Therefore, 15P(90C10A) fabricated in an air bath formed an interconnected bi-continuous symmetrical structure, and the membrane had bigger pore sizes than membrane fabricated in a water bath, as shown in Figure 8.

### 2.4. The Permeability and Porosity of Membranes

**Figure 9:**
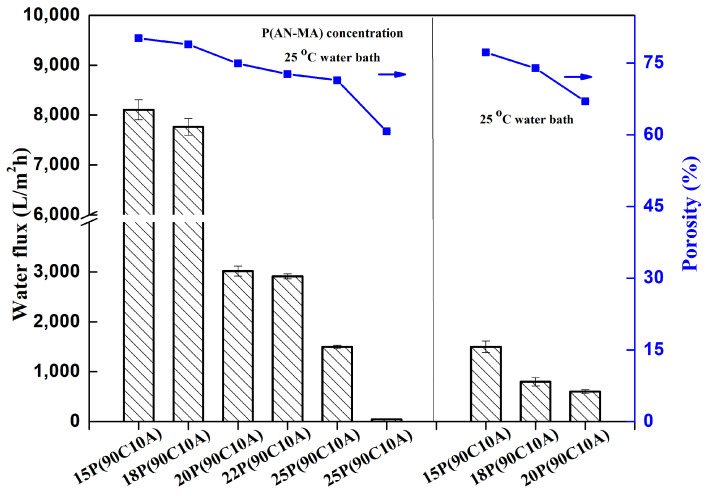
Water flux (bars) and porosity (squares) of PAN-based microfiltration membranes prepared under various conditions.

The water flux measurements of P(AN-MA) membranes fabricated at different P(AN-MA) concentrations and cooling environments, as presented in Figure 9, reveal notable trends. It is observed that the water flux increases as the P(AN-MA) concentration and cooling rate decrease, aligning with the interconnected sponge structures and porous surface characteristics described in Figure 2 and Figure 4. Specifically, the membrane prepared from 15P(90A10C) in a 25 °C air bath exhibited the highest water flux (8107 L/m^2^·h), which was attributed to its largest porosity (81.0%) and pore size (0.25 μm).When the P(AN-MA) concentration was increased from 15% to 30% and fabricated in an air bath, the membrane experienced a decrease in pore size and porosity, leading to a decrease in the water flux from 8107 L/m^2^·h to 49 L/m^2^·h. Similarly, the membrane composed of 15P(90C10A) and prepared in a 25 °C water bath experienced a reduction in the water flux, which fell to approximately 1498 L/m^2^·h.

These findings underscore the significant impact of P(AN-MA) concentration and cooling environments on the permeability of membranes, which can be attributed to the porosity, connectivity, and pore size of the membranes. Ultimately, it can be concluded that larger pore size, greater porosity, and enhanced connectivity contribute to higher water flux.

**Figure 10:**
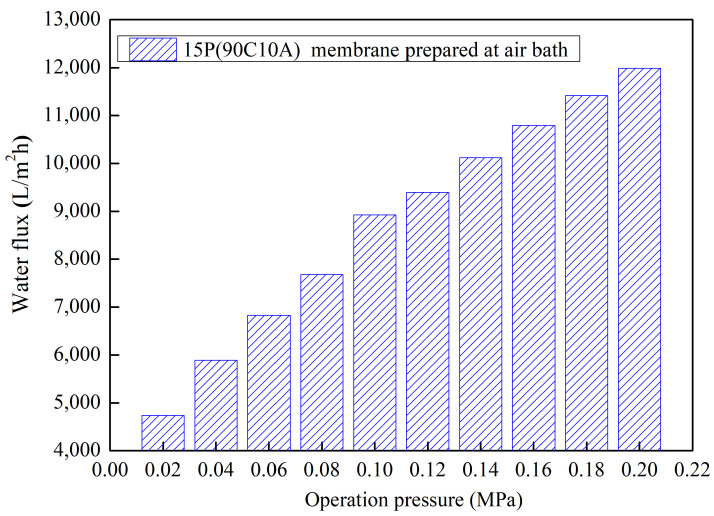
The pure water permeation of 15P(90C10A) membrane fabricated in a 25 °C air bath with increased pressure.

The evaluation of pure water flux for the 15P(90C10A) membrane at different operating pressures ranging from 0.02 MPa to 0.2 MPa with a gradient of 0.02 MPa, as depicted in Figure 10, yielded significant insights. The results illustrate a nearly linear increase in water flux with progressive operation pressure. Specifically, as the operation pressure increased from 0.02 MPa to 0.2 MPa, the corresponding water flux values were measured at 4739 L/m^2^·h and 11,988 L/m^2^·h, respectively.

These findings provide compelling evidence of the membrane’s good stress resistance, as the symmetrical structure demonstrates an ability to accommodate higher operating pressures while maintaining a proportional increase in water flux. This characteristic is crucial for ensuring the reliability and performance of the membrane during filtration processes conducted under varying pressure conditions.

### 2.5. Wettability of Membranes

The P(AN-MA) membrane contained hydrophobic groups (-COOCH_3_) and hydrophilic groups (-CN), resulting in a higher water contact angle (115°) and a lower oil contact angle (0°) compared to those of traditional PAN membranes, respectively. When the membrane was submerged in water, its underwater contact angles towards various types of oil (including petroleum ether, hexane, and toluene) were all approximately 140°, demonstrating underwater hydrophobicity. When fully soaked with oil, its water contact angles in carbon tetrachloride exceeded 148°, indicating underoil hydrophobicity.

**Figure 11:**
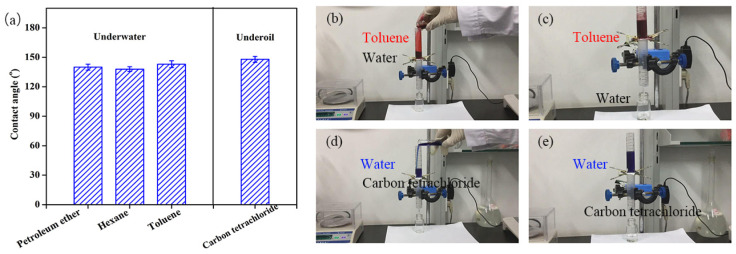
(**a**) Underwater oil contact angle and underoil water contact angle. Oil/water mixture separation tests using 15P(90C10A) membrane. (**b**,**c**) The membrane was first wetted by water before the separation process and separated oil/water mixture ($\rho_{Oil}<\rho_{Water}$). (**d**,**e**) The membrane was first wetted by water before the separation process and separated oil/water mixture ($\rho_{Oil}<\rho_{Water}$).

To confirm the adaptable wettability of the P(AN-MA) membrane for on-demand oil/water separation, experiments were conducted using various oil/water mixtures. As depicted in Figure 11b,c, when a mixture of toluene and water was introduced into the separation system, the oil phase (dyed red) was repelled by the underwater hydrophobic membrane, allowing water to permeate the membrane and be collected. When the membrane was pre-wetted with oil before the separation under the same conditions, oil selectively permeated through the membrane while water was retained (Figure 11d,e).

### 2.6. Oil/Water Mixture Separation and the Tensile Strength of Membranes

**Figure 12:**
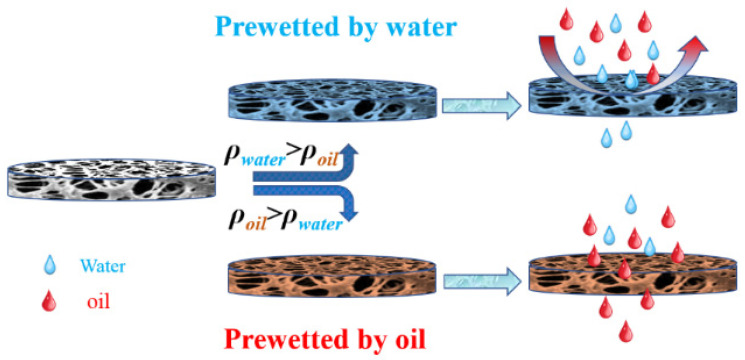
Schematic illustration of the selective separation of oil/water mixtures.

The comprehensive investigation of the separation performance of P(AN-MA) membranes for immiscible oil/water mixtures, including petroleum ether/water, toluene/water, hexane/water, and carbon tetrachloride/water mixtures, is a significant advancement. The ability of the P(AN-MA) membrane to separate immiscible oil/water mixtures under gravity. They were poured onto the separation system and pre-wetted by water or oil, respectively, as demonstrated in Figure 12.

**Figure 13:**
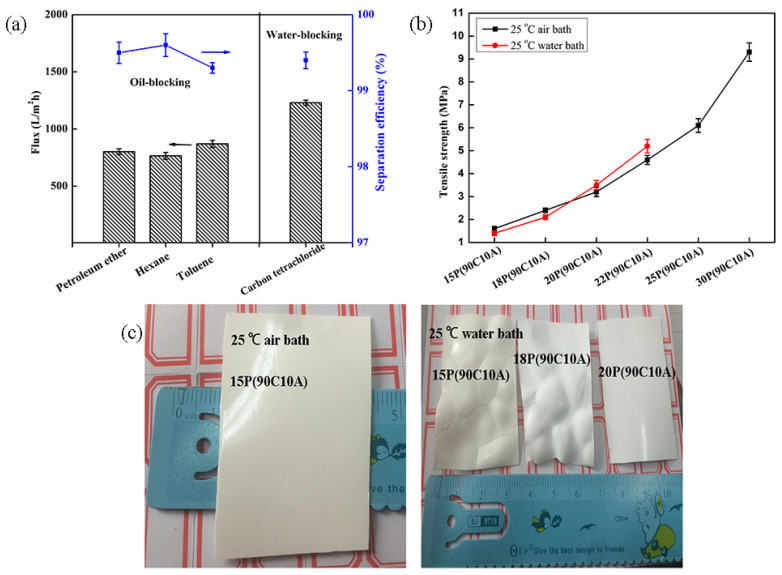
(**a**) Water or oil fluxes and the corresponding separation efficiency in the filtrate from an oil/water mixture using 15P(90C10A) membranes under gravity. (**b**) Tensile strength of the wet P(AN-MA) membranes. (**c**) Photograph of P(AN-MA) membranes dried in air.

The pure water flux of petroleum ether/water mixture was 870 L/m^2^ h and the pure oil flux of petroleum tetrachloride/water mixture was 1230 L/m^2^ h under gravity (Figure 13a). The tensile strength and water fluxes showed an opposite changing trend, as shown in Figure 13b. The tensile strength of wet membranes increased with the polymer content. This was associated with the decrease in membrane porosity and pore size. In the air bath, the membranes’ tensile strength showed an obvious improvement from 1.9 MPa to 9.3 MPa, when the P(AN-MA) content increased from 15 wt.% to 30 wt.%. Although 15(90C10A) and 18(90C10A) fabricated in a water bath had smaller pore size and porosity than membrane fabricated in an air bath, these membranes had lower tensile strength.

Membranes prepared in a pair of quartz glasses mold (1.04 mm, thick) as shown in Figure 5, induced only a vertical temperature gradient from the membrane solution to the bottom and the top of the mold. For low P(AN-MA) content with a fast cooling rate, microcracks and defects easily occurred on the surfaces of the membranes, which was due to the poor control of the temperature gradient (Figure 13c). These defects severely affect the tensile strength of membrane. However, in the water bath, when the P(AN-MA) content was increased to 20 wt.%, the microcracks and defects in the membranes disappeared. This can be explained by the increase in viscosity. As a result, 20P(90C10A) prepared in the water bath had microcrack-free, perfect surface, a smaller pore size, and lower porosity than that fabricated in an air bath, which had a better tensile strength.

### 2.7. Reliability of the P(AN-MA) Membrane and Recovery of Crystallizable Solvent

**Figure 14:**
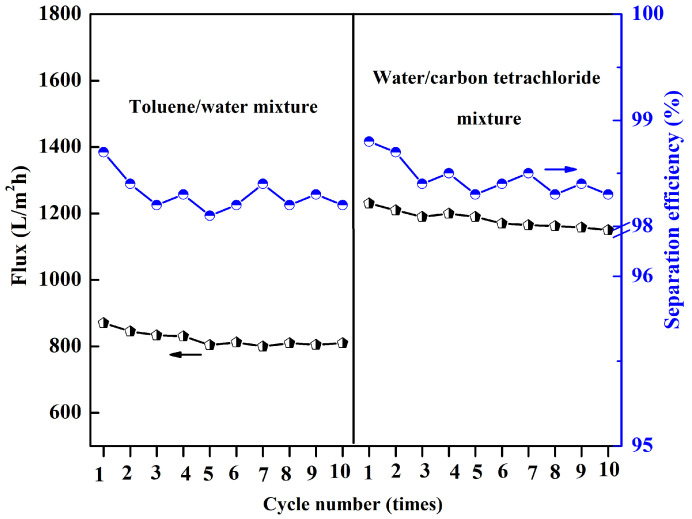
The cycling separation performance of the P(AN-MA) membrane was evaluated for toluene/water and water/carbon tetrachloride mixtures. The black and blue line indicated flux and seperation efficiency, respectively.

The reliability of the P(AN-MA) membrane was further assessed, as this is an important consideration for practical applications. The membrane maintained its high separation efficiency even after 10 cycles of separation. During each cycle, the membrane was simply washed with ethanol and pure water several times. It was found that the separation efficiency remained above 98.0%, and the water or oil flux values only exhibited a slight decrease after 10 cycles of filtration, as depicted in Figure 14.

**Figure 15:**
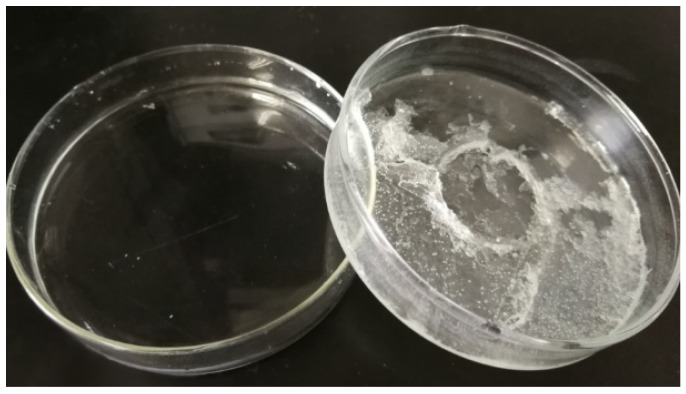
Recovery results of mixed diluent from the nascent PAN-based microfiltration membranes.

The recovery process of CPL/CA by recrystallization from water, along with the extraction of diluent solvents from the as-prepared membranes placed in 25 °C deionized water, offers an environmentally friendly and cost-effective approach. The obtained CPL/CA aqueous solution was concentrated at 60 °C and then cooled at 25 °C for 24 h, resulting in the recovery of CPL/CA diluent solvents, achieving an impressive recovery efficiency of 85.3%, as depicted in Figure 15.

This streamlined recovery method involving recrystallization presents significant advantages, as it simplifies the process, reduces potential pollution, and ultimately cuts down production costs. These outcomes hold promising implications for sustainable and efficient processes within the membrane fabrication industry.

## 3. Materials and Methods

### 3.1. Materials

The melt-processable P(AN-MA) copolymer was synthesized. Caprolactam (CPL, 99%), acetamide (AC, 98%), methylene blue, and oil red were purchased from Aladdin. Deionized (DI) water obtained from a Millipore MilliQ system was used as an extractant. P(AN-MA) copolymer was dried at 60 °C under vacuum for 2 h before use, and the other chemicals were used without further purification. 

### 3.2. Fabrication of P(AN-MA) Membranes

P(AN-MA) and diluents were mixed together in a closed glass vessel, which was heated at 140 °C in a nitrogen atmosphere for 3 h until a homogeneous solution was formed with a mechanical stirrer. When the air bubbles were degassed, the solution was quickly transferred into a pair of quartz glass molds (200 × 200 mm) to form a film (thickness ~150 μm), then the mold was put into the oven at 140 °C for 5 min. Subsequently, the mold was immediately immersed in a cooling bath (an air or water bath at 25 °C) to induce phase separation. When the nascent membrane was totally solidified, it was taken out of the mold, and the as-prepared membranes were placed in a 25 °C deionized water and ethyl alcohol bath to extract the diluent solvents. The compositions and preparation conditions of each membrane are listed in Table 2.

### 3.3. Phase Separation Behavior

The phase separation process of P(AN-MA)/CPL/AC was obtained by optical microscopy (Olympus, BX51, Tokyo, Japan). The cloud point (T_cloud_) was observed when the turbidity appeared during the cooling process. In order to prevent diluent loss, a Teflon ring was used between the two microscope slides. The solid sample was heated to 140 °C at 20 °C/min, maintained for 2 min to eliminate the thermal history, and then cooled to room temperature at 5 °C/min.

The melting points (T_m_) and the crystallization temperatures (T_c_) of the P(AN-MA)/CPL/AC mixtures were measured using a differential scanning calorimeter (Model DSC200F3, Netzsch, Selb, Germany). A 5~8 mg sample was put into an aluminum pan, which was heated from 25 °C to 140 °C at 20 °C/min and maintained at 140 °C for 2 min to eliminate the thermal history, cooled from 140 °C to 0 °C at 10 °C/min, and then reheated to 140 °C at 10 °C/min under the protection of a nitrogen atmosphere. 

The viscosity of the P(AN-MA)/CPL/AC ternary system was determined by (HAAKE MARS, Karlsruhe, Germany). The temperature was scanned from 160 °C to 80 °C and the cooling rate was 10 °C/min.

### 3.4. Characterization of the P(AN-MA) Membranes

The surface and cross-sectional morphologies were observed by FESEM (Hitachi S-4800, Hitachi, Ibraraki, Japan) at an acceleration voltage of 10.0 kV. P(AN-MA) samples were frozen and fractured in liquid nitrogen to obtain a tidy cross-section. Then, the samples were sputtered with gold using a sputter coater (SCD-005, Leica Microsystems, Wetzlar, Germany).

The tensile strength of the samples was measured by a tensile testing machine (CMT4503, Meitesi Industry Co. Ltd., Shenzhen, China). The pore size was measured by the nuclear magnetic method (PDNMR20-015V, Suzhou niumag analytical corporation, Suzhou, China).

### 3.5. Properties of Membranes

The overall porosity of P(AN-MA) membranes was calculated by the following formula:

(1) $$P=\frac{\frac{m_{1}-m_{2}}{\rho_{water}}}{\frac{m_{1}-m_{2}}{\rho_{water}}+\frac{m_{2}}{\rho_{\mathrm{AN-MA}}}}\times 100\%$$

where m_1_ and m_2_ are the weight of the wet and dry P(AN-MA) membrane, respectively. ρ_AN-MA_ is the density of P(AN-MA), and ρ_water_ is the density of water at 25 °C (1.0 g/cm^3^).

The pure water permeability of membranes was determined by a homemade device with an active area of 4.9 cm^2^. First, the membranes were pre-wet with pure water and pre-pressurized at 0.12 MPa, which was maintained at 25 °C for 30 min until the flux recorded as J_W_ was stable. Then, the transmembrane pressure was reduced to 0.10 MPa to measure the membrane flux. To determine the stress of the membrane, we measured the permeate flux by increasing the operation pressure from 0.02 to 0.2 MPa with a gradient of 0.02 MPa. The J_w_ was defined by the following formula:

(2) $$J_{w}=\frac{V}{A\times ∆t}$$

where J_w_ is the pure water flux (L/m^2^.h), V is the volume of permeated water (L), A is the available membrane area (m^2^) and Δt is the permeate time (h). 

### 3.6. Separation Experiment for Immiscible Oil/Water Mixtures

The organic solvents (including petroleum ether, toluene, hexane, and carbon tetrachloride) were dyed with oil red. The water was colored by CuSO_4_. They were mixed (1:1, *v*/*v*) to form petroleum ether/water mixture, toluene/water mixture, hexane/water mixture, and carbon tetrachloride/water mixture, respectively. They were poured onto the separation system, which was pre-wetted with water or oil, respectively.

## 4. Conclusions

The successful fabrication of P(AN-MA) membranes via the TIPS method has yielded remarkable results. These membranes exhibit an interconnected sponge structure and porous surface, particularly when the content of AC is 10 wt.% in a 25 °C air bath, demonstrating excellent permeability with a pure water flux of up to 8107 L/m^2^h and a porosity of up to 81.0 wt.%. This outstanding performance enables the highly efficient separation of immiscible oil/water mixtures, as evidenced by the pure water flux of 870 L/m^2^.h for the petroleum ether/water mixture and the pure oil flux of 1230 L/m^2^.h for the petroleum tetrachloride/water mixture under gravity.

Additionally, the recovery efficiency of diluents reached 85.3% through recrystallization, offering significant environmental and cost-saving advantages, thus reducing potential pollution and production costs.

These findings collectively underscore the promising potential of P(AN-MA) membranes for practical applications in various separation processes, while also highlighting the environmentally conscious and economically favorable aspects of their fabrication and recovery processes.

## Figures and Tables

**Table 1:** Solubility parameters of P(AN-MA), CPL and AC.

Materials	δ_d_ (MPa^0.5^)	δ_p_ (MPa^0.5^)	δ_h_ (MPa^0.5^)	δ (MPa^0.5^)	Δδ_12_ (MPa)	Ref.
P(AN-MA)	19.49	10.77	8.26	23.75		[23]
CPL	19.30	10	11.6	24.60	11.78	[32]
AC	17.30	18.7	22.4	33.92	267.6	[37]

δ, δ_d_, δ_p_, and δ_h_ are the overall solubility, dispersion, polar and hydrogen parameters, respectively. Δδ_12_ = [(δ_d1_ − δ_d2_)^2^ + (δ_p1_ − δ_p2_)^2^ + (δ_h1_ − δ_h2_)^2^]^1/2^, where 1 and 2, respectively, denote the polymer and solvent.

**Table 2:** Preparation conditions of P(AN-MA) membranes.

Sample	P(AN-MA) Concentration (%)	CPL Mass Fraction in the Mixed Diluent (%)	Cooling Bath (°C)
15P(90C10A)	15	90	25 ^a^
18P(90C10A)	18	90	25 ^a^
20P(90C10A)	20	90	25 ^a^
22P(90C10A)	22	90	25
25P(90C10A)	25	90	25
30P(90C10A)	30	90	25

^a^ The cooling media were a water and air bath at 25 °C and another air bath at 25 °C. The ingredients were denoted as xP(yCzA), where P, C, and A represent P(AN-MA), CPL, and AC, respectively.

## Data Availability

Data are contained within the article.
